# Deep sequencing and pathway-focused analysis revealed multigene oncodriver signatures predicting survival outcomes in advanced colorectal cancer

**DOI:** 10.1038/s41389-018-0066-2

**Published:** 2018-07-22

**Authors:** Francesca De Nicola, Frauke Goeman, Matteo Pallocca, Francesca Sperati, Laura Pizzuti, Elisa Melucci, Beatrice Casini, Carla Azzurra Amoreo, Enzo Gallo, Maria Grazia Diodoro, Simonetta Buglioni, Marco Mazzotta, Patrizia Vici, Domenico Sergi, Luigi Di Lauro, Maddalena Barba, Edoardo Pescarmona, Gennaro Ciliberto, Ruggero De Maria, Maurizio Fanciulli, Marcello Maugeri-Saccà

**Affiliations:** 1 SAFU Laboratory, Department of Research, Advanced Diagnostic, and Technological Innovation, IRCCS “Regina Elena” National Cancer Institute, Via Elio Chianesi 53, 00144 Rome, Italy; 2 Oncogenomic and Epigenetic Unit, IRCCS “Regina Elena” National Cancer Institute, Via Elio Chianesi 53, 00144 Rome, Italy; 3 Biostatistics-Scientific Direction, IRCCS “Regina Elena” National Cancer Institute, Via Elio Chianesi 53, 00144 Rome, Italy; 4 Division of Medical Oncology 2, IRCCS “Regina Elena” National Cancer Institute, Via Elio Chianesi 53, 00144 Rome, Italy; 5 Department of Pathology, IRCCS “Regina Elena” National Cancer Institute, Via Elio Chianesi 53, 00144 Rome, Italy; 6 Medical Oncology Unit, Policlinico Sant’Andrea, Via Di Grotta Rossa 1035/1039, 00189 Rome, Italy; 7 Scientific Direction, IRCCS “Regina Elena” National Cancer Institute, Via Elio Chianesi 53, 00144 Rome, Italy; 8 Institute of General Pathology, Catholic University of the Sacred Heart, Largo Agostino Gemelli, 10, 00168 Rome, Italy

## Abstract

Genomic technologies are reshaping the molecular landscape of colorectal cancer (CRC), revealing that oncogenic driver mutations (*APC* and *TP53*) coexist with still underappreciated genetic events. We hypothesized that mutational analysis of CRC-linked genes may provide novel information on the connection between genetically-deregulated pathways and clinical outcomes. We performed next-generation sequencing (NGS) analysis of 16 recurrently mutated genes in CRC exploiting tissue specimens from 98 advanced CRC patients. Multiple correspondence analysis (MCA) was used to identify gene sets characterizing negative and positive outliers (patients in the lowest and highest quartile of progression-free survival, PFS). Variables potentially affecting PFS and overall survival (OS) were tested in univariate and multivariate Cox proportional hazard models. Sensitivity analyses and resampling were used to assess the robustness of genomic predictors. MCA revealed that *APC* and *TP53* mutations were close to the negative outlier group, whereas mutations in other WNT pathway genes were in proximity of the positive outliers. Reasoning that genetic alterations interact epistatically, producing greater or weaker consequences in combination than when individually considered, we tested whether patients whose tumors carried a genetic background characterized by *APC* and *TP53* mutations without coexisting mutations in other WNT genes (*AMER1*, *FBXW7*, *TCF7L2*, *CTNNB1*, *SOX9*) had adverse survival outcomes. With this approach, we identified two oncodriver signatures (ODS1 and ODS2) associated with shorter PFS (ODS1 multivariate Cox for PFS: HR 2.16, 95%CI: 1.28–3.64, *p* = 0.004; ODS2 multivariate Cox for PFS: HR 2.61, 95%CI: 1.49–4.58, *p* = 0.001). Clinically-focused and molecularly-focused sensitivity analyses, resampling, and reclassification of mutations confirmed the stability of ODS1/2. Moreover, ODS1/2 negatively impacted OS. Collectively, our results point to co-occurring driver mutations as an adverse molecular factor in advanced CRC. This relationship depends on a broader genetic context highlighting the importance of genetic interactions.

## Introduction

Central in the appreciation of colorectal cancer (CRC) pathogenesis was the identification of the adenoma-carcinoma sequence, a model describing the stepwise acquisition of mutations in master regulators of cell fate, growth, and differentiation such as *APC*, *TP53*, and *KRAS*^1–3^. A second key step towards understanding CRC biology was the identification of a subset of tumors, accounting for ~10–15% of all cases, characterized by microsatellite instability (MSI)^4–6^. MSI stems from epigenetic inactivation or germline mutations in the DNA mismatch repair (MMR) machinery, giving rise to replication infidelity and a hypermutated phenotype^7^. More recently, large genomic characterization efforts helped elucidate the molecular landscape of CRC highlighting that, beyond established oncogenic drivers, a number of other genes are frequently altered^7–10^. In 2012, the Cancer Genome Atlas Research (TCGA) Network provided the largest catalogue of recurrently altered genes in CRC, conveying the message that, independent from microsatellite status, genetic derangements converge into, and perturb, a fairly limited number of molecular circuits^7^. Indeed, integrative pathway analysis delineated a molecular scenario dominated by alterations in: (i) intestinal stem cell pathways (WNT and TGF-β), (ii) PI3K signaling, (iii) RAS-MAPK cascade, and (iv) p53-mediated control of cell-cycle checkpoints and apoptosis^7^.

Over the past decade, the advent of molecularly targeted agents fueled a wave of investigations striving to assess the clinical exploitability of common genetic events in CRC^11^. Beyond the *RAS* status (*KRAS* and *NRAS*) that is routinely tested for the administration of EGFR-directed therapy^12^, great expectations surround the possibility of turning off oncogenic PI3K and BRAF signalings^13,14^, whereas MMR deficiency has recently been connected with sensitivity to programmed death 1 (PD-1) inhibition^15,16^. While, on the one hand, the availability of targeted agents along with the granularity reached by high-throughput genomic technologies are streamlining the developmental path of novel compounds in selected patient’ populations, on the other hand the clinical significance of many recurrently mutated genes in CRC still remains unclear.

On this ground, we applied targeted DNA next-generation sequencing (NGS) to assess the mutational status of frequently mutated genes in tissue samples from 98 advanced CRC (sporadic) patients treated with first-line therapy. In particular, we focused on 16 genes altering the following signaling avenues: WNT (*AMER1*, also known as *WTX* or *FAM123B*, *APC*, *CTNNB1*, *FBXW7*, *SOX9*, *TCF7L2*), TGF-β (*ACVR1B*, *SMAD2*, *SMAD4*), PI3K (*PIK3CA* and *PTEN*), RAS/MAPK (*BRAF*, *MAP3K21*, also known as *KIAA1804*, *KRAS*, *NRAS*) and cell-cycle/apoptosis (*TP53*). The choice of the aforementioned genes is rooted in the molecular characterization of CRC carried out by the TCGA network^7^. Indeed, 14 out of the 16 evaluated genes were those identified as recurrently mutated in the non-hypermutated setting. *BRAF* was considered in light of its clinical relevance, whereas *PTEN* was included due to its connection with the *PI3K* pathway and oncogene-induced replication stress. Through this biology-driven approach we sought to identify genomic predictors of survival outcomes.

## Results

### Baseline characteristics of the patients and mutational profile

**Fig. 1:**
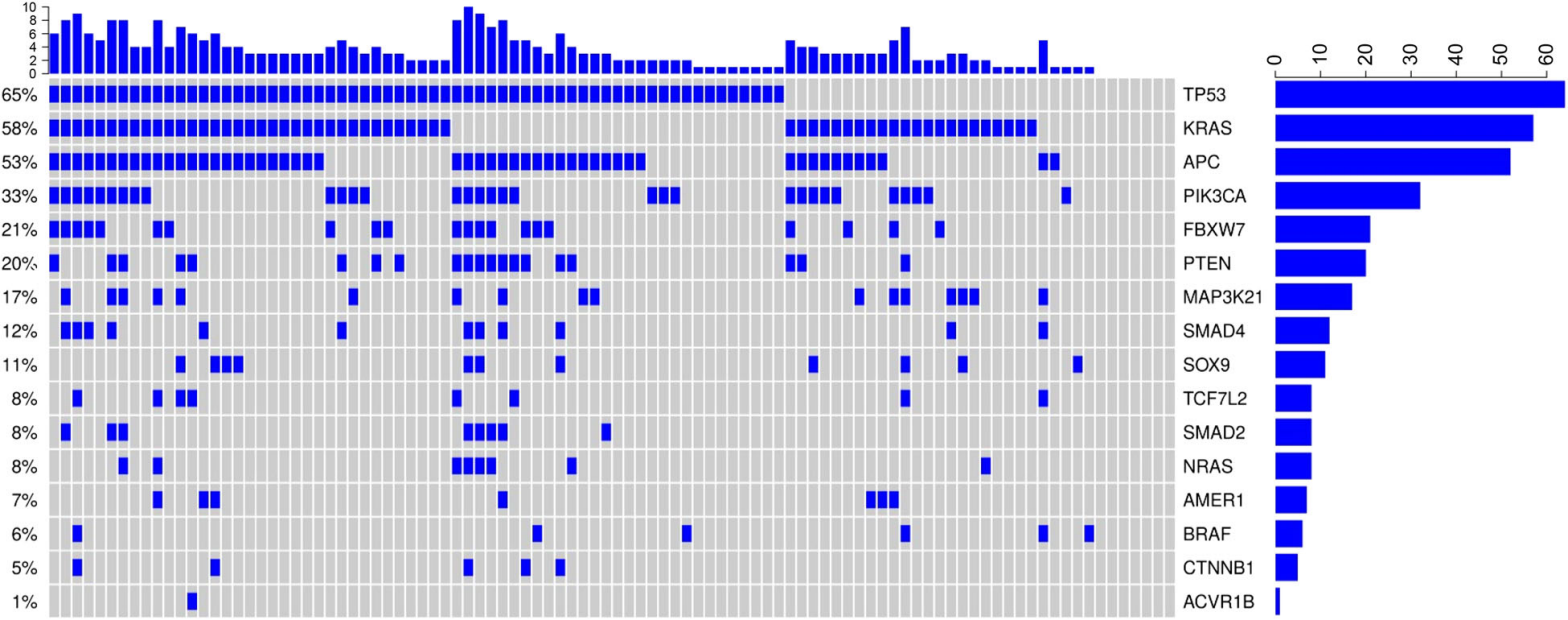
Oncoprint showing the distribution of mutations in 16 CRC-related genes. Samples with mutations are indicated in blue

Baseline characteristics of the 98 patients included in the present study are summarized in Table 1. In the first-line setting, 20 patients (20.4%) received chemotherapy plus cetuximab, 19 patients (19.4%) received chemotherapy plus bevacizumab, and 59 patients (60.2%) were treated with chemotherapy alone. In this cohort, 29 patients (29.6%) underwent surgery for metastatic disease, and three of them achieved a R0 resection. The mutational rates of the 16 CRC-related genes is illustrated in Supplementary Table 1, which also provides a comparison with four publically available datasets (TCGA, DFCI, MSKCC, Genentech, available at http://www.cbioportal.org/)^7,8,10,17^. Overall, our results are consistent with those reported by independent investigators. The individual distribution of mutations is illustrated in Fig. 1, whereas a detailed overview of the detected mutations is presented in Supplementary Figure 1. Significant associations between the investigational biomarkers and basal clinical features are provided in Supplementary Table 2.

**Table 1:** Baseline characteristics of colorectal cancer (CRC) patients included in this study (*N* = 98)

Characteristics		*N* (%)
Age at diagnosis	Median [IQ range]	61.6 [55.2–69.9]
Gender	Male	63 (64.3)
	Female	35 (35.7)
Stage at diagnosis	II–III	34 (34.7)
	IV	64 (65.3)
(Neo)Adjuvant therapy	No	72 (73.5)
	Yes	26 (26.5)
ECOG PS	0	47 (48.0)
	1–2	51 (52.0)
Side	Right	32 (32.7)
	Transverse	11 (11.2)
	Left	55 (56.1)
Number of metastatic sites	1	52 (53.1)
	≥2	46 (46.9)
Surgery for metastatic disease	No	69 (70.4)
	Yes	29 (29.6)
First-line therapy	Chemotherapy^a^	59 (60.2)
	Chemotherapy/Cetuximab^b^	20 (20.4)
	Chemotherapy/Bevacizumab^c^	19 (19.4)
Second-line therapy	No	31 (31.6)
	Yes	67 (68.4)
Targeted agent	No	44 (44.9)
	Yes (first-line and beyond)	54 (55.1)

^a^FOLFIRI *N* = 39, FOLFOX *N* = 20

^b^FOLFIRI/Cetuximab *N* = 14, FOLFOX/Cetuximab *N* = 5, CPT-11/Cetuximab *N* = 1

^c^FOLFIRI/Bevacizumab *N* = 14, FOLFOX/Bevacizumab *N* = 4, Capecitabine/Bevacizumab *N* = 1

### Multiple correspondence analysis (MCA) revealed gene sets associated with positive/negative outliers

**Fig. 2:**
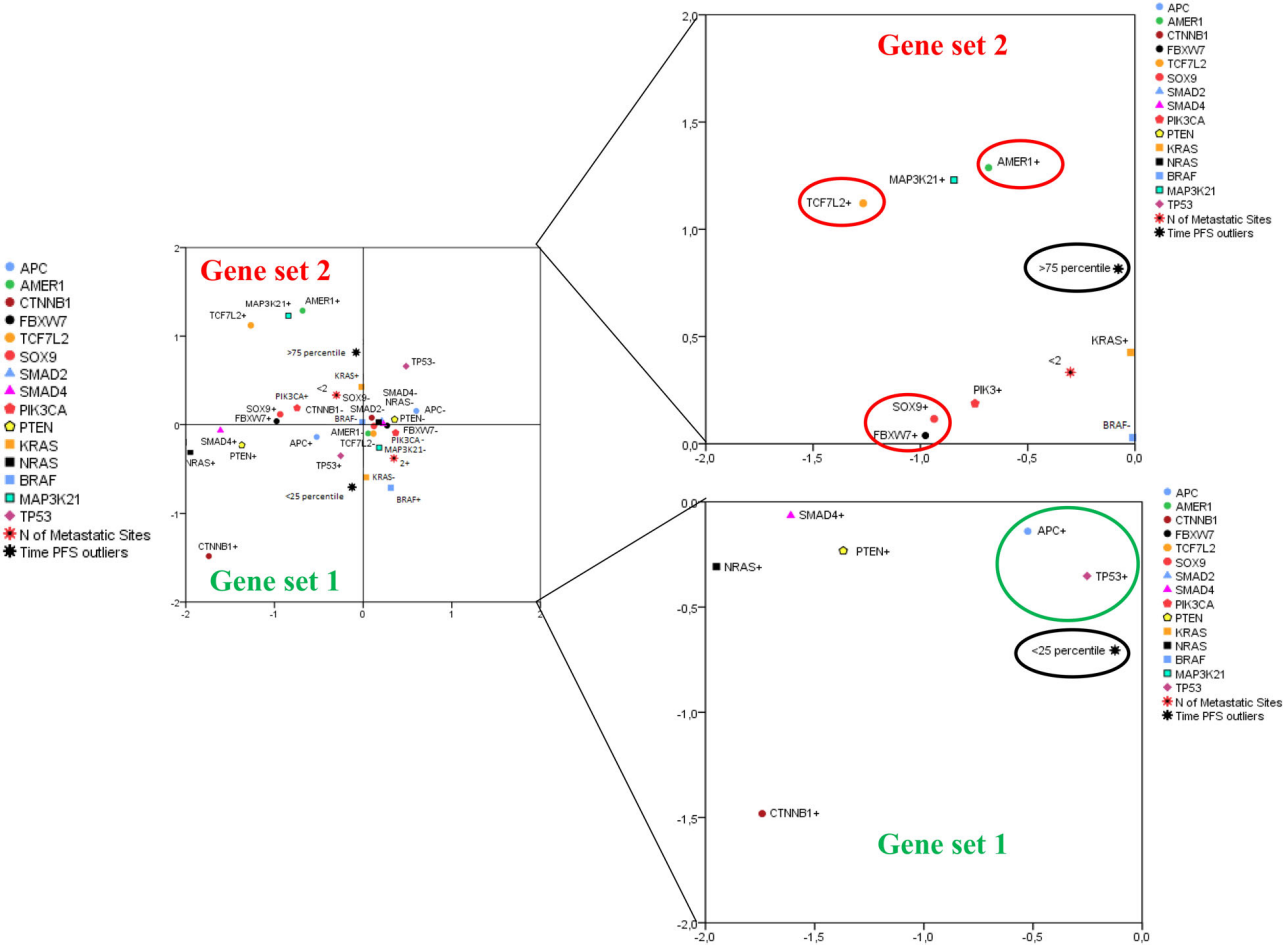
Multiple correspondence analysis (MCA, leftmost panel) graphically depicting the relationship between the mutational status of 16 CRC-linked genes and positive/negative outliers for PFS (see text for details). Mutations in *APC* and *TP53* (gene set 1, larger view in the lower rightmost panel) are close to the negative outlier group (black circle and green circle), whereas mutations in various WNT pathway genes (gene set 2, larger view in the upper rightmost panel) are close to the positive outlier group (black circle and red circles). Mutant genes are indicated with (+), whereas the wild-type form with (−)

When individually considered, none of the evaluated genes was significantly associated with shorter progression-free survival (PFS), with the exception of *BRAF*^*V600E*^ mutations (4/98) that conferred shorter PFS (log-rank *p* = 0.001, data available upon request). Reasoning that the connection between CRC-related mutations and clinical outcomes might be dependent on a specific mutational repertoire instead of a single event, we performed MCA to obtain an overview of the mutational landscape potentially characterizing good responders (positive outliers) and poor responders (negative outliers). As detailed in the “Statistical analyses” section, these two groups were obtained by considering patients with the longest (positive outliers) and shortest (negative outliers) PFS. MCA indicated that a series of mutant genes was located nearby the negative outliers (Fig. 2). This gene set (gene set 1, GS1) was characterized by the presence of mutant *TP53* and *APC*, two established oncogenic drivers in CRC. A second set of mutant genes was close to the positive outliers (gene set 2, GS2), and was enriched for the presence of mutations in other WNT pathway components, namely *AMER1*, *TCF7L2*, *FBXW7*, *SOX9* (total variance: 29.7%, dimension 1: 19.8%, dimension 2: 9.9%).

### Identification of oncogenic driver-based signatures predicting shorter PFS

**Fig. 3:**
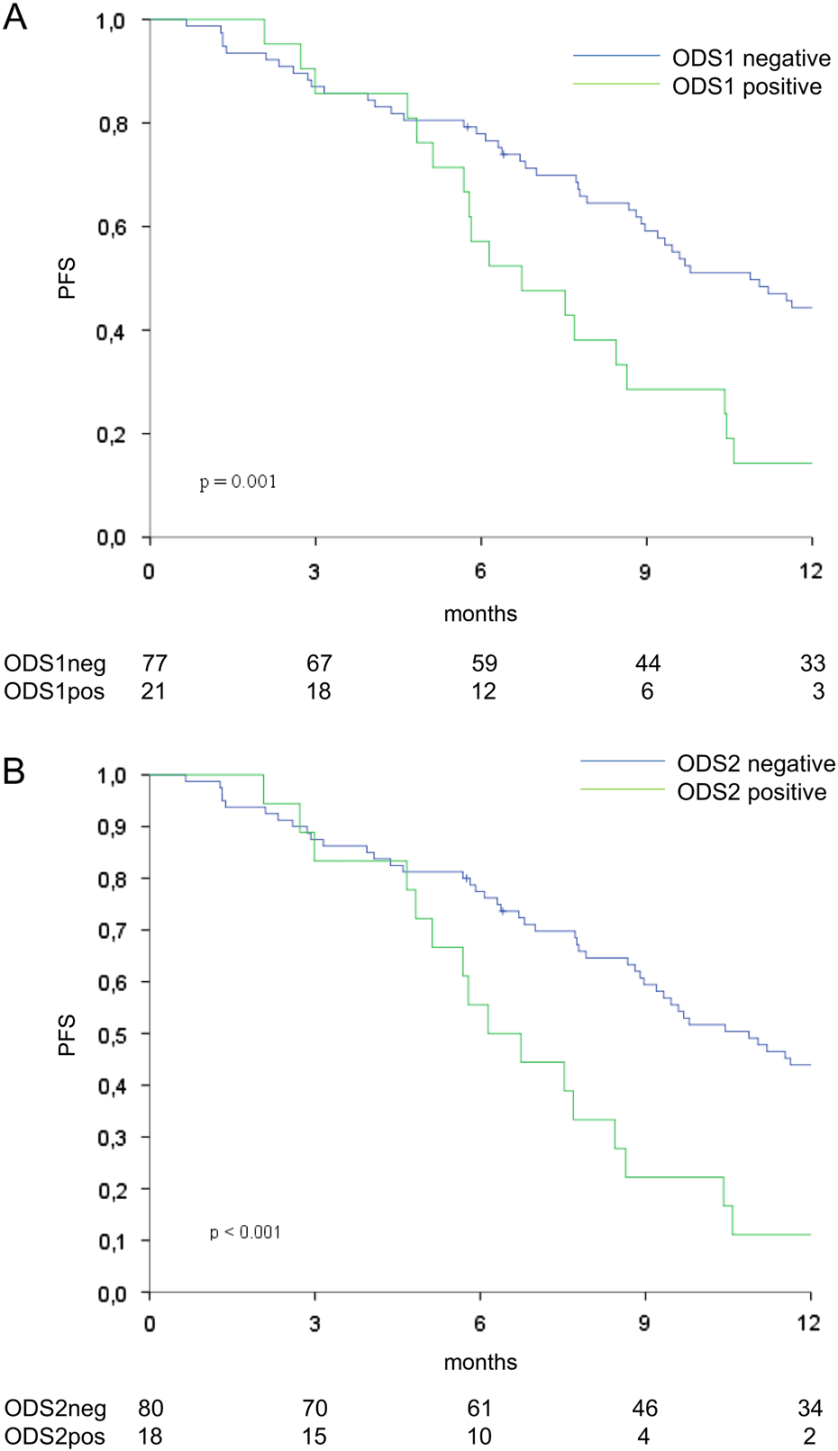
Kaplan-Meier survival curves of progression-free survival comparing ODS1-positive vs. negative cases (**a**) and ODS2-positive cases vs. their negative counterparts (**b**) (*N* = 98)

The results from MCA prompted us to hypothesize that while the co-occurrence of *TP53* and *APC* mutations (GS1) plausibly reflects a more aggressive molecular portrait, mutations in less-frequently mutated genes (GS2) might partly soften the deleterious effects of driver mutations (negative genetic interactions, deleterious passengers)^18,19^. Moreover, while a mutation in a given pathway confers a survival advantage, a dual mutational hit on the same signaling avenue may negatively impact cell fitness. On this basis, we verified whether the co-occurrence of *TP53* and *APC* mutations (oncogenic drivers present in GS1), in association with the wild-type form of WNT pathway genes represented in the GS2, negatively impacted PFS. Consistently with this hypothesis, we observed that patients whose tumors harbored the aforementioned characteristics (*TP53*mut/*APC*mut/*AMER1*wt/*TCF7L2*wt/*FBXW7*wt, OncoDriver signature 1, ODS1, *N* = 21/98) had significantly shorter PFS (log-rang *p* = 0.001; Fig. 3a). Comparable results were obtained when introducing wild-type *SOX9* and *CTNNB1* (β-catenin) in the model (*N* = 18/98) (OncoDriver signature 2, ODS2: log-rang *p* < 0.001, Fig. 3b). Thus, ODS2 encompasses the entire set of WNT pathway genes (*TP53*mut/*APC*mut/*AMER1*wt/*TCF7L2*wt/*FBXW7*wt/*SOX9*wt/*CTNNB1*wt) (Supplementary Table 3). Univariate and multivariate Cox regression models for PFS (Table 2 and Supplementary Table 4) confirmed that patients whose tumors harbored these signatures were at increased risk of disease progression (ODS1 multivariate Cox adjusted for variables testing significant at univariate analyses: HR 2.16, 95%CI: 1.28–3.64, *p* = 0.004. ODS2 multivariate Cox adjusted for variables testing significant at univariate analyses: HR 2.61, 95%CI: 1.49–4.58, *p* = 0.001) (Table 2). Results from the multivariate Cox models indicate that ODS1/2 are independent predictors of an increased risk of disease progression, given that the presence of other factors potentially affecting PFS (i.e., type of therapy, disease burden, performance status) did not alter their adverse clinical significance.

**Table 2:** Univariate and multivariate Cox regression models for progression-free survival (*N* = 98)

		Univariate Cox regression model		Multivariate Cox regression model (ODS1 pos vs neg)		Multivariate Cox regression model (ODS2 pos vs neg)	
		HR (95% CI)	*p*-value	HR (95% CI)	*p*-value	HR (95% CI)	*p*-value
ODS1	Pos vs Neg	2.42 (1.44–4.06)	0.001	2.16 (1.28–3.64)	0.004		
ODS2	Pos vs Neg	2.76 (1.59–4.80)	<0.001			2.61 (1.49–4.58)	0.001
Gender	Female vs Male	0.79 (0.51–1.24)	0.315				
ECOG PS	1–2 vs 0	1.25 (0.82–1.89)	0.293				
Side	DX + TV vs SX	0.93 (0.61–1.41)	0.730				
Targeted Agents in first-line therapy	Yes vs No	0.86 (0.57–1.31)	0.498				
Surgery for metastatic disease	Yes vs No	0.63 (0.40–1.01)	0.054	0.58 (0.36–0.93)	0.024	0.56 (0.35–0.89)	0.015
Number of metastatic sites	2–3 vs 1	1.75 (1.14–2.68)	0.010	1.73 (1.11–2.70)	0.016	1.81 (1.16–2.82)	0.009

The multivariate Cox models were built with variables testing significant in univariate analysis

### Robustness of ODS1 and ODS2

**Fig. 4:**
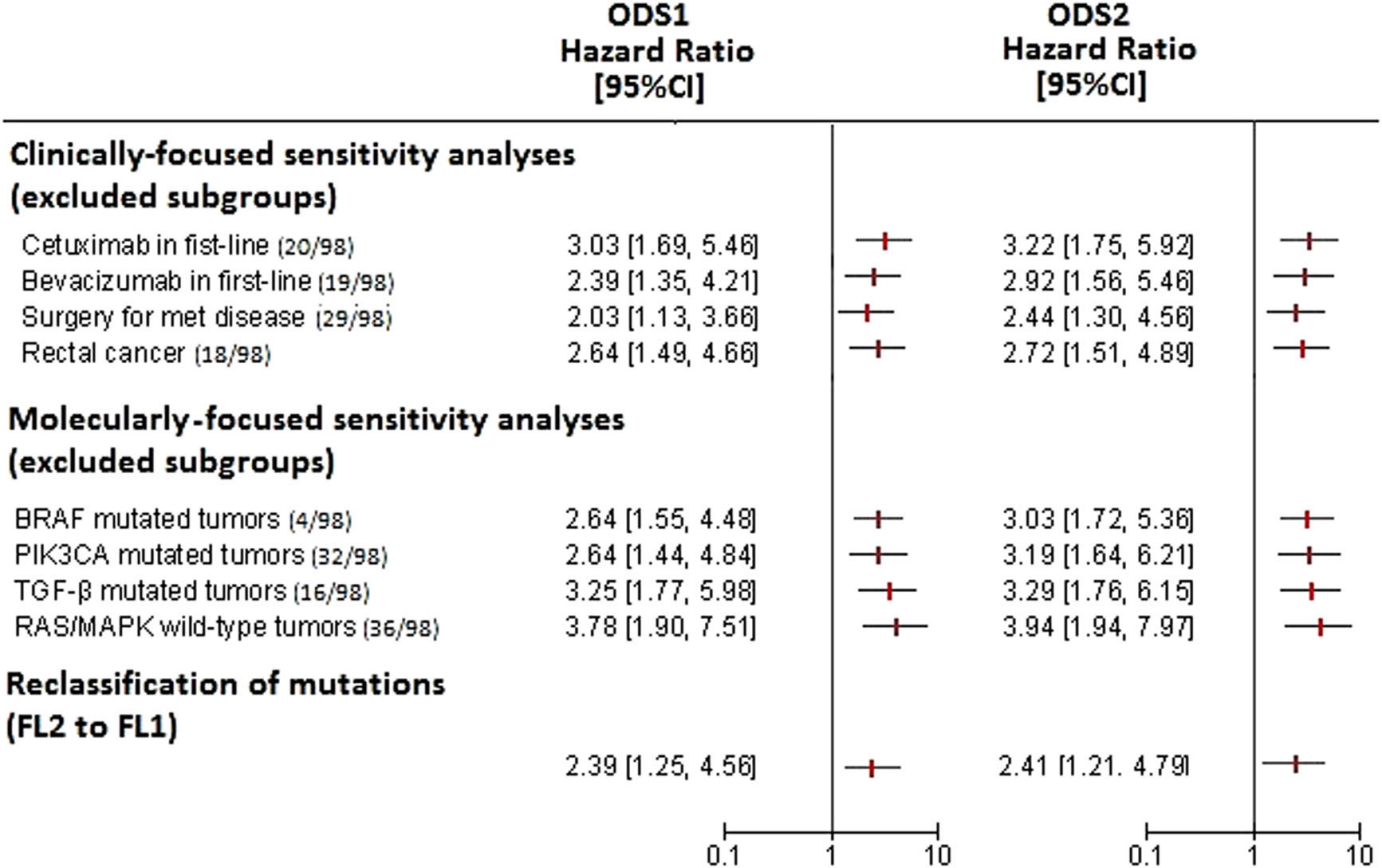
Forest plot illustrating univariate Cox regression analyses (ODS1 and ODS2) for progression-free survival. From top to bottom: clinically-focused sensitivity analyses, molecularly-focused sensitivity analyses, reclassification of mutations (FL2 to FL1)

In order to assess the stability of ODS1 and ODS2, we performed a wave of analyses based on the following procedures: (i) sensitivity analyses (exclusion of specific clinically and molecularly relevant subgroups), (ii) adoption of a less stringent criterion for assessing mutations (switch from FL2 to FL1, as detailed in the “Next-generation sequencing” section), and (iii) random resampling without replacement (detailed in the “Statistical analyses” section). The entire battery of sensitivity analyses confirmed the relationship between ODS1/2 and PFS (Fig. 4). Thus, the increased risk of progression conferred by ODS1/2 is independent of the treatment administered, the surgical removal of metastatic lesions, as well as of the presence or absence of mutations in other frequently-deregulated pathways.

Comparable results were obtained upon reclassifying mutations exploiting FL1, thus without taking into account their established or predicted functional consequence on the encoded protein (Fig. 4). Finally, upon resampling, the replication rate for the univariate Cox model was 93 and 99% for ODS1 and ODS2, respectively, with statistical significance set at *p* < 0.01. This indicates that ODS1/2 retain their significance even when evaluated in less-powered cohorts.

### ODS1 and ODS2 are associated with inferior overall survival (OS)

We next investigated whether ODS1/2 conferred an increased risk of death. Patients whose tumors carried ODS1/2 had shorter OS, independently whether OS was computed from the first cycle of chemotherapy or from diagnosis (ODS1: log-rank *p* = 0.005 and *p* = 0.004, respectively, Supplementary Figure 2, panel A and B; ODS2: log-rank *p* = 0.003 for both, Supplementary Figure 2, panel C and D). The multivariate Cox regression models for OS (Table 3 and Supplementary Table 5) indicated that the two signatures were associated with an increased risk of death (ODS1: multivariate Cox adjusted for variables testing significant at univariate analyses: HR 2.14, 95%CI: 1.19–3.82, *p* = 0.011. ODS2: multivariate Cox adjusted for variables testing significant at univariate analyses: HR 2.36, 95%CI: 1.28–4.35, *p* = 0.006) (Table 3). Thus, ODS1/2 negatively impacted both PFS and OS.

**Table 3:** Univariate and multivariate Cox regression models for overall survival (*N* = 98)

		Univariate Cox regression model		Multivariate Cox regression model (ODS1 pos vs neg)		Multivariate Cox regression model (ODS2 pos vs neg)	
		HR (95%CI)	*p*-value	HR (95%CI)	*p*-value	HR (95%CI)	*p*-value
ODS1	Pos vs Neg	2.21 (1.26–3.88)	0.006	2.14 (1.19–3.82)	0.011		
ODS2	Pos vs Neg	2.34 (1.31–4.20)	0.004			2.36 (1.28–4.35)	0.006
Gender	Female vs Male	0.79 (0.47–1.33)	0.369				
Stage at diagnosis	IV vs II-III	1.24 (0.74–2.08)	0.412				
ECOG PS	1–2 vs 0	2.12 (1.28–3.52)	0.003	1.85 (1.09–3.15)	0.023	1.79 (1.05–3.05)	0.032
Side	DX + TV vs SX	1.07 (0.66–1.76)	0.777				
Second-line therapy	Yes vs No	0.29 (0.17–0.48)	<0.001	0.22 (0.12–0.39)	<0.001	0.21 (0.12–0.38)	<0.001
Surgery for metastatic disease	Yes vs No	0.58 (0.34–0.99)	0.046	0.48 (0.27–0.84)	0.010	0.48 (0.27–0.85)	0.012
Number of metastatic sites	2–3 vs 1	1.40 (0.85–2.33)	0.188				
Targeted agents	Yes vs No	0.66 (0.40–1.07)	0.094				

The multivariate Cox models were built with variables testing significant at univariate analysis

## Discussion

In the present study, we analyzed the mutational status of 16 CRC-linked genes in a cohort of 98 CRC patients with advanced disease treated with first-line therapy. This study capitalizes on consolidated evidence that assigns to *TP53* and *APC* mutations a central role in colorectal carcinogenesis, along with recent data from large molecular characterization initiatives that are helping to elucidate the genetic landscape of CRC^1,7–10^. Collectively, our results suggest that: (i) a subset of CRC is characterized by a genomic signature denoting concomitant deregulation of *TP53* and *APC*, and (ii) the coexistence of *TP53* and *APC* mutations predicts shorter PFS and OS only in the absence of mutations in other genes collocated in the WNT signaling, and that also intersect the p53 network at the protein level. To our knowledge, this is the first report exploiting mutational profiling to identify multigene predictors of PFS in the advanced setting. This hindered comparable analyses in publically available datasets (e.g., TCGA). Indeed, other databases do not contain the necessary information to verify our findings including PFS and complete data on administered chemotherapy throughout the natural history of the disease.

In interpreting our results, we acknowledge that some points necessitate clarification. First, we exploited FFPE tissues, which predispose to artifacts. For this reason, we sought to be as stringent as possible in determining the mutational status of the 16 investigated genes, limiting our attention to mutational events already described in 16 TCGA datasets (obtained from frozen tissue specimens using paired tumor/normal data)^7^. With this approach, the mutational rates and patterns were comparable to what was observed by independent research groups^7,8,10,17^. Second, microsatellite status was available for 22 samples (data not shown), and none of these displayed microsatellite instability. Evidence that the predictive role of ODS1/2 was unaffected by excluding specific patient subgroups (sensitivity analyses), even patients whose tumors carried the *BRAF*^*V600E*^ mutation, refrained us from pursuing this further level of characterization.

Earlier reports did not specifically focus on the advanced disease setting^9,20^, or aimed at evaluating the genomic concordance between primary and matched metastatic tumors^10^. More specifically, Schell et al.^9^ have recently reported that *APC* mutations are associated with worse prognosis only in the presence of *KRAS* and *TP53* mutations. Our data in the metastatic setting extend these findings, adding novel information that can foster the development, validation and implementation of genomic predictors. Indeed, we focused on PFS as the primary outcome measure, which represents the most direct indicator of efficacy/inefficacy of anticancer agents. Importantly, the straightforward analytical approach carried out for challenging the consistency of the two signatures did not modify their predictive ability. Clinically-driven and molecularly-driven sensitivity analyses conveyed the message that the adverse significance of ODS1/2 was unrelated to both treatment-related features and deregulation of other signaling transduction pathways. Indeed, the link between ODS1/2 and an increased risk of tumor progression was maintained when excluding patients with specific characteristics potentially impacting PFS, such as those who received targeted agents (cetuximab or bevacizumab) in association with chemotherapy in the first-line setting, or those who underwent surgical excision of metastatic lesions. This was further enforced by the results deriving from multivariate Cox regression models, where ODS1/2 tested statistically significant independently from the number and nature of variables included in the models. In other words, the negative significance of ODS1/2 was independent of a series of other factors that may confound the interpretation of the results if not properly considered. Likewise, the resampling procedure indicated the stability of ODS1/2 across underpowered, randomly-generated datasets. This indicates the robustness of ODS1/2, as their predictive ability was not modified when running analyses in cohorts smaller in size. Finally, ODS1/2 remained associated with shorter PFS even when re-classifying mutant samples with the adoption of FL1.

Recent advances in understanding the genetic interactions that propel tumor progression and therapeutic resistance assisted us in framing our results. It is known that while a mutation in a given pathway confers a survival advantage to cancer cells, the concomitant presence of another genetic event in the same signaling cascade may be detrimental for cell viability^21^. This phenomenon is therapeutically exploited in the search for lethal interactions^22^. Moreover, passenger events, traditionally viewed as neutral, are nowadays considered capable of reducing proliferative fitness and metastatic progression (“deleterious passengers”)^19^. In addition, while the link between *APC* and the other WNT pathway genes herein evaluated is intuitive, mechanistic studies have also tied these genes to the p53 network. For instance, WTX (AMER1) modulates p53 activity through enhancing CBP/p300-mediated p53 acetylation^23^, whereas FBXW7 was identified as a mediator of the p53 response to DNA damage^24^. Thus, the detrimental effect on cell viability elicited by multiple mutations in the same pathway/function, and/or by passenger mutations, plausibly explains why co-existing *TP53* and *APC* mutations are associated with poorer survival outcomes exclusively in the absence of other genetic events in functionally connected genes. Likewise, these type of genomic interactions may account for the stability of ODS1/2 when the pathogenic significance of mutations was not considered (FL1). In turn, the idea that driver genetic events require a low mutational burden to fully express their oncogenic repertoire might be exploited when pursuing the pharmacological targeting of APC and p53. Regarding ODS2, we acknowledge that this predictor reclassifies only a limited number of patients when compared to ODS1 (*N* = 18 vs 21). Nevertheless, ODS2 deserves consideration for two intertwined reasons. First, multivariate Cox regression models indicate a further increase in the risk of progression and death with this more complex multigene predictor. Second, ODS2 provides further ground to our hypothesis, namely the detrimental effect of an excessive mutational load, especially when the target is the same pathway/function.

A further aspect that requires some comments is the collocation of *PIK3CA* and *CTNNB1* mutations in the MCA. In doing so, it must be considered that MCA is exploratory by nature, being exclusively intended to provide clues on whether one or more variables might be associated with the outcome of interest. In our analysis, *PIK3CA* mutations are located in proximity of the positive outliers, despite they are widely perceived as an important oncogenic force in CRC^25^. Nevertheless, a recent systematic review and meta-analysis including 28 studies for a total of 12,747 patients showed that *PIK3CA* mutation has neutral prognostic effects, as it did not significantly impact clinical outcomes^26^. Our results are consistent with this meta-analysis and with current evidence on the impact of specific mutations on clinical outcomes. Indeed, in our cohort *PIK3CA* mutations were neither associated with PFS nor with OS, whereas the only predictor that, when individually considered, conferred adverse survival outcomes was the *BRAF*^*V600E*^ mutation (data available upon request)^14^. These same considerations apply to *CTNNB1*, for which we considered the wild-type form in building ODS2, despite mutant β-catenin was located nearby the negative outliers in the MCA plot. The logic behind this stems from the detrimental effects on cell viability elicited by a dual mutations hits on the same pathway, given that *APC* mutations are central for the performances of ODS1/2^21,22^.

The strategy we are adopting to achieve a rapid and efficient translation of this knowledge into the clinical setting deserves a final mention. As a general principle, this is centered on an extensive pathway-level analysis, both at the clinical and preclinical level. First, we are expanding our biorepository of clinically-annotated samples for molecular analyses, with the goal of doubling the current cohort. Second, protein-level and transcript-level characterization of genetically-deregulated molecular networks has been initiated. In a first instance, we focused on signaling communicating with the WNT pathway in the control of intestinal stem cell fate, and with the p53-mediated orchestration of the DNA damage response. Such extensive characterization is being coupled with mechanistic studies by exploiting a collection of patient-derived CRC stem cells (CRC-SCs)^27,28^. Thus, our final goal is twofold. First, optimizing the signatures herein identified by integrating the information retrieved at various level of characterization (gene, transcript, and protein) in a larger case series. This is instrumental for the prospective part of this study with biomarker validation purposes, having provided the necessary information, such as the frequency of candidate biomarkers and effect difference between positive and negative cases. Second, we will strive to identify investigational or established compounds capable of selectively eliminating CRC-SC carrying specific molecular backgrounds, thus providing a sound rationale for biomarker-driven trials.

Overall, our data point to mutations in oncogenic drivers as plausible predictors of survival outcomes in advanced CRC patients treated with systemic therapy in the advanced setting. This relationship depends on a wider genetic context, as it requires the absence of mutations in genes belonging to the WNT signaling.

## Materials and methods

### Patients

For this analysis, 98 patients with histologically confirmed, metastatic CRC who received first-line chemotherapy with or without targeted agents between September 2000 and September 2016 were included. Median follow-up was 22 months (IQR 11–34 months). Patients were considered eligible if complete data on clinical features, treatment outcomes, and mutational profiling were available. Tumor responses were evaluated by Response Evaluation Criteria in Solid Tumors (RECIST) criteria v.1.1. PFS was calculated as the time between the first cycle of chemotherapy until radiological evidence of disease progression or death due to any cause. OS was computed as the time from the first cycle of chemotherapy to death due to any cause, and as the time from diagnosis to death due to any cause. This study was conducted in accordance with the Declaration of Helsinki and approved by the Ethics Committee of the “Regina Elena” National Cancer Institute of Rome. Written informed consents were obtained by all the participants. This study adheres to the REMARK guidelines^29^.

### Next-generation sequencing

For targeted DNA resequencing, we considered tissue samples collected before the administration of systemic therapies for advanced disease. All specimens were reviewed for histological verification and to ensure a tumor content > 50%. Genomic DNA was extracted from 5 µm FFPE tissue sections using the AllPrep DNA/RNA FFPE kit (Qiagen, Valencia, CA, USA). To perform the targeted DNA resequencing, a custom panel employing DesignStudio from Illumina was designed. The TruSeq Custom Amplicon Kit was used for library preparation. Samples were sequenced on an Illumina NextSeq 500 (Illumina, Inc., San Diego, CA, USA) in paired-end mode, sequencing from each side 150 bp. Primary analysis encompassing FASTQ file generation, alignment and variant calling was performed on the Illumina BaseSpace Cloud environment, using the Truseq Amplicon analysis pipeline version 2.0. TSV files were generated from VCFs with the Illumina Variant Studio software version 3.0. Low-coverage (<200×), dbSNP annotated variants (MAF > 5%), mutations with a variant allele frequency (VAF) < 5%, and mutations that were not found in 16 final TCGA datasets were filtered out (colorectal adenocarcinoma, stomach adenocarcinoma, esophagus-stomach cancers, head and neck squamous cell carcinoma, non-small cell lung cancer including adenocarcinoma and squamous cell carcinoma, invasive breast carcinoma, uterine corpus endometrial carcinoma, ovarian serous cystadenocarcinoma, clear cell renal carcinoma, chromophobe renal cell carcinoma, bladder urothelial carcinoma, prostate adenocarcinoma, papillary thyroid carcinoma, glioblastoma multiforme, acute myeloid leukemia). We referred to this filtering procedure as filtering level 1 (FL1). Finally, we also considered the established or predicted pathogenicity of the detected mutations, by exploiting OncoKB and Mutation Assessor accessed via cBioPortal Version v1.8.3 (last accessed on 25 October 2017). This procedure was defined as filtering level 2 (FL2). FL2 represented the main classification procedure in the identification of genomic signatures associated with survival outcomes, whereas FL1 was employed to assess their robustness. Investigators who performed NGS analysis were blinded to treatment outcomes (PFS and OS).

### Statistical analyses

Descriptive statistics were computed for all the variables of interest. The relationship between categorical variables was investigated with the Pearson’s Chi-squared test of independence (two-tailed) or the Fisher Exact test, depending on the size of the groups compared. MCA was exploited to uncover the relationship between the mutational status of 16 CRC-associated genes (wild-type and mutated) and negative/positive outliers, defined as patients in the lowest (PFS < 5.8 months) and highest (PFS > 14.9 months) quartile for PFS. Similarity was measured as chi-squared distance. Survival curves were estimated with the Kaplan-Meier product-limit method and compared by log-rank test. Variables potentially affecting PFS and OS were tested in univariate Cox proportional hazard models. Multivariate Cox models were built by adjusting for variables testing significant at the univariate analysis, and also by including all the variables assessed in univariate analyses. The related estimates were reported as hazard ratio (HR) and 95% confident interval (CI). To assess the robustness of genetic backgrounds associated with PFS, we conducted a number of clinically- and molecularly-focused sensitivity analyses by excluding the following subgroups: cetuximab in the first-line setting (*N* = 20/98), bevacizumab in the first-line setting (*N* = 19/98), surgery for metastatic disease (*N* = 29/98), rectal cancer (*N* = 18/98), *BRAF*^*V600E*^ mutated tumors (*N* = 4/98), *KRAS*/*NRAS/BRAF* triple wild-type tumors (*N* = 36/98), *PIK3CA* mutated tumors (*N* = 32/98), and TGF-β mutated tumors (*N* = 16/98), defined as tumors carrying at least one mutation in *SMAD2*, *SMAD4* or *ACRVB1*. Level of significance was defined as *p* < 0.05. The consistency of genetic signatures was further evaluated through a procedure envisioning re-sampling without replacement^30,31^. More specifically, one hundred, less-powered datasets were generated by randomly removing ~20% of the original sample. For each simulation, the univariate Cox model was repeated and the replication rate was calculated, with statistical significance set at *p* < 0.01. Statistical analyses were carried out using SPSS version 21.0 (SPSS Inc., Chicago, Illinois, USA).

## Electronic supplementary material

Supplementary Figure 1-2 and supplementary tables 1-5
