# Supplementary material for: Deep sequencing and pathway-focused analysis revealed multigene oncodriver signatures predicting survival outcomes in advanced colorectal cancer
Source: Oncogenesis. 2018 Jul 22;7(7):55. doi: 10.1038/s41389-018-0066-2 (PMC6054833; doi:10.1038/s41389-018-0066-2)
Supplement: Supplementary file 1 — Supplementary Figure 1-2 and supplementary tables 1-5 [file 41389_2018_66_MOESM1_ESM.pdf]

Supplementary Figure 1: MutationMapper illustrating the detected mutations (and their nature) represented on the linear proteins.

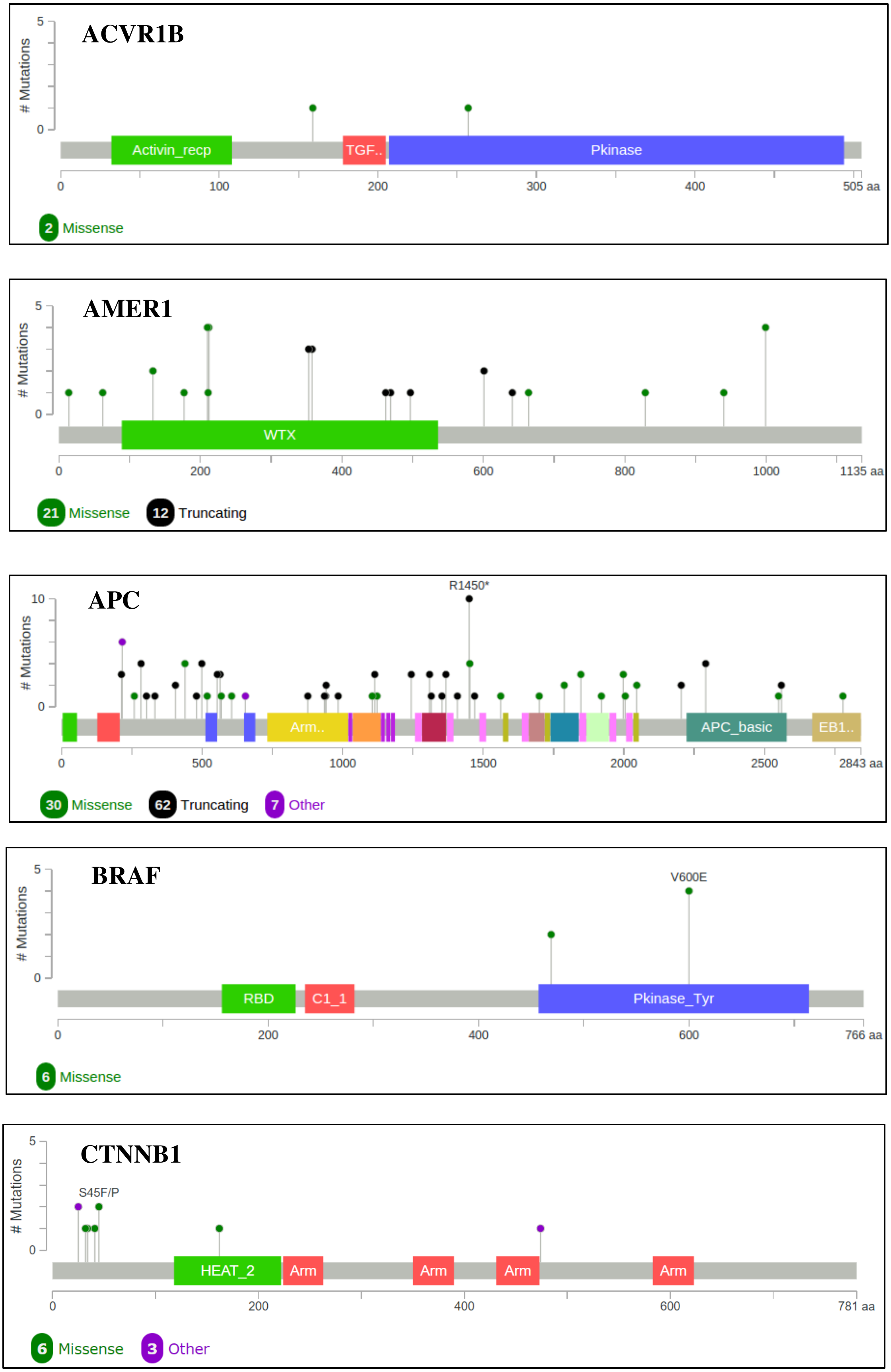

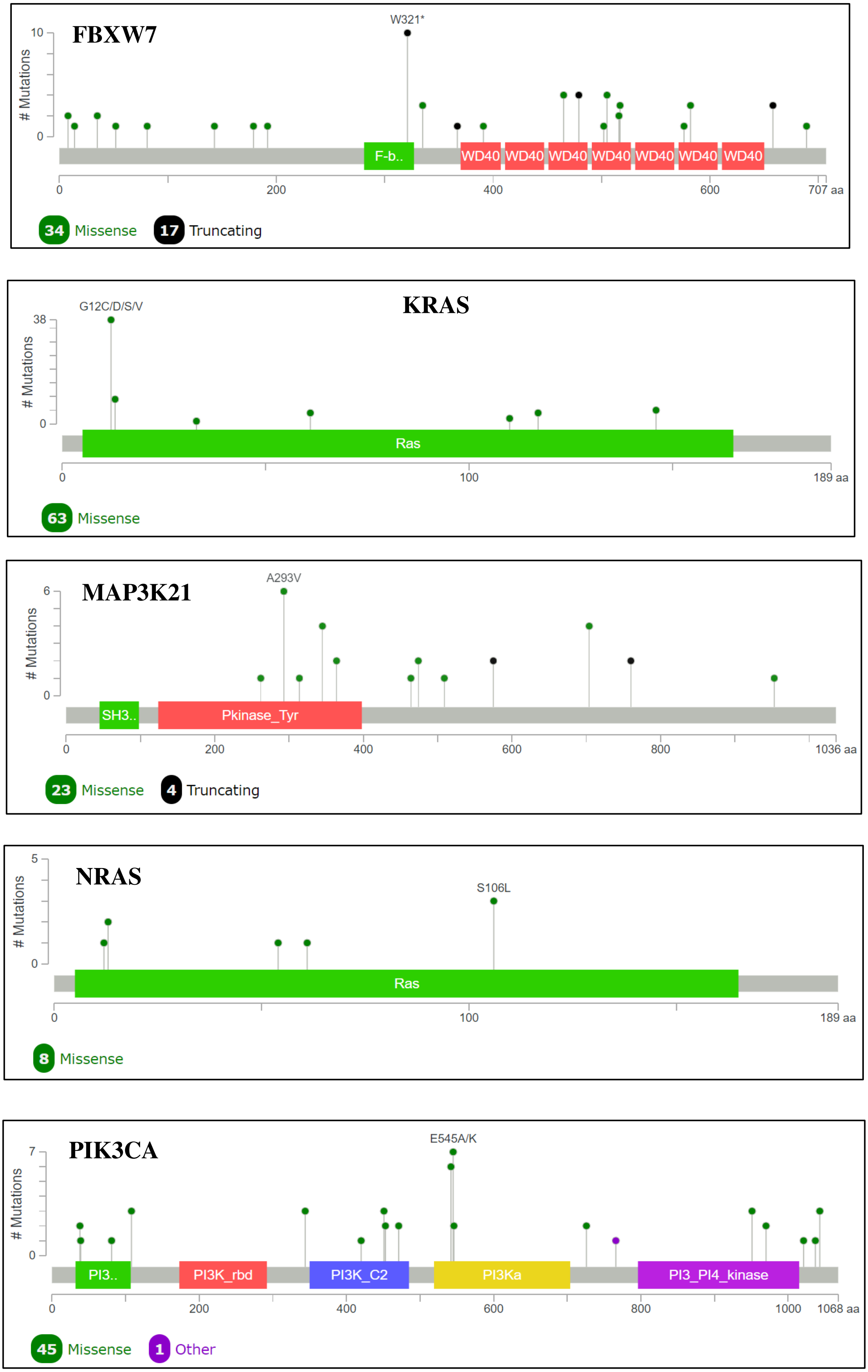
FBXW7
W321*
Mutations
10
0
F-b..
WD40
WD40
WD40
WD40
WD40
WD40
WD40
0
200
400
600
707 aa
34 Missense
17 Truncating
KRAS
G12C/D/S/V
Mutations
38
0
Ras
0
100
189 aa
63 Missense
MAP3K21
A293V
Mutations
6
0
SH3..
Pkinase_Tyr
0
200
400
600
800
1036 aa
23 Missense
4 Truncating
NRAS
S106L
Mutations
5
0
Ras
0
100
189 aa
8 Missense
PIK3CA
E545A/K
Mutations
7
0
PI3..
PI3K_rbd
PI3K_C2
PI3Ka
PI3_PI4_kinase
0
200
400
600
800
1000
1068 aa
45 Missense
1 Other

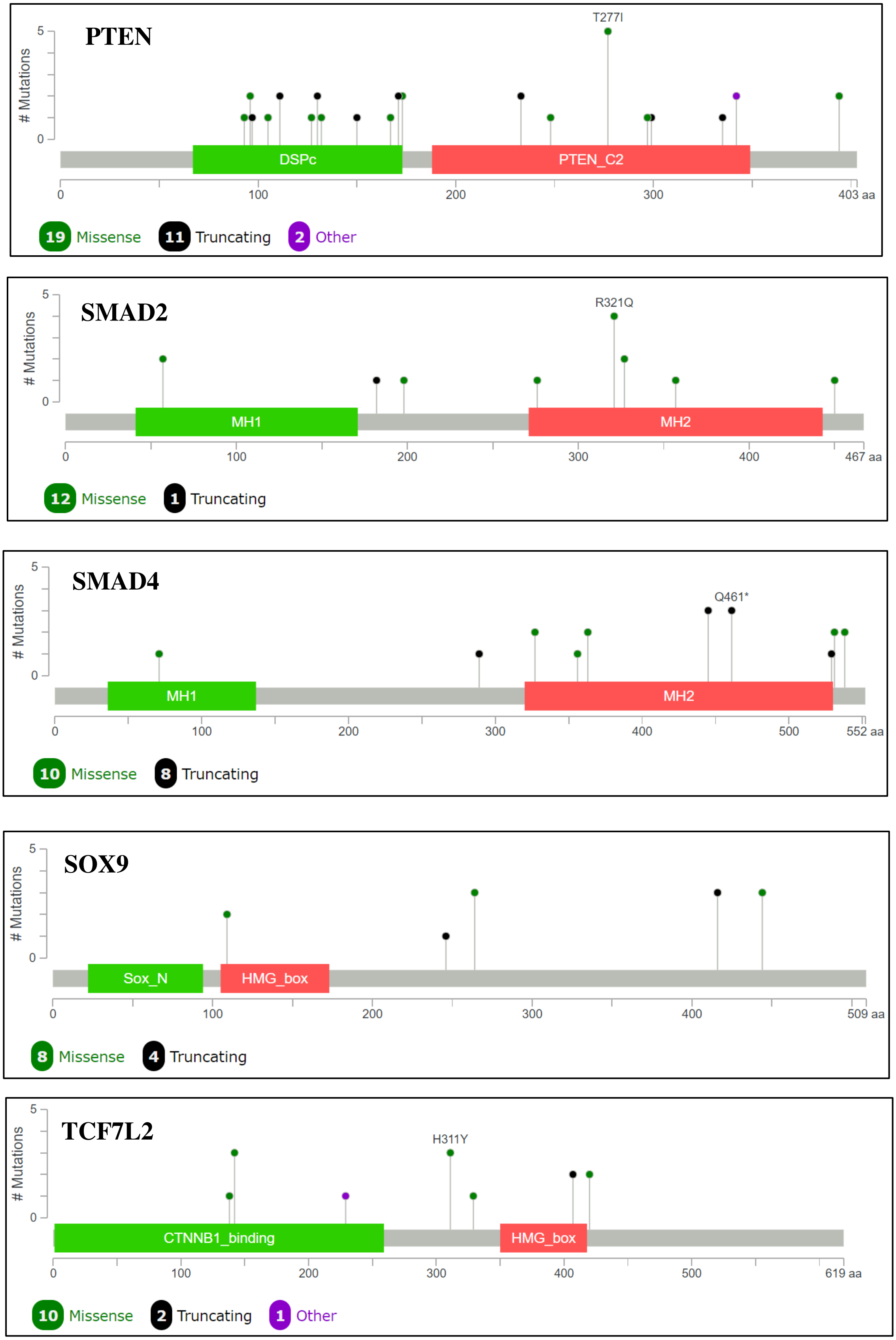
PTEN
Mutations
5
0
T277I
DSPc
PTEN_C2
0
100
200
300
403 aa
19 Missense
11 Truncating
2 Other
SMAD2
Mutations
5
0
R321Q
MH1
MH2
0
100
200
300
400
467 aa
12 Missense
1 Truncating
SMAD4
Mutations
5
0
Q461*
MH1
MH2
0
100
200
300
400
500
552 aa
10 Missense
8 Truncating
SOX9
Mutations
5
0
Sox_N
HMG_box
0
100
200
300
400
509 aa
8 Missense
4 Truncating
TCF7L2
Mutations
5
0
H311Y
CTNNB1_binding
HMG_box
0
100
200
300
400
500
619 aa
10 Missense
2 Truncating
1 Other

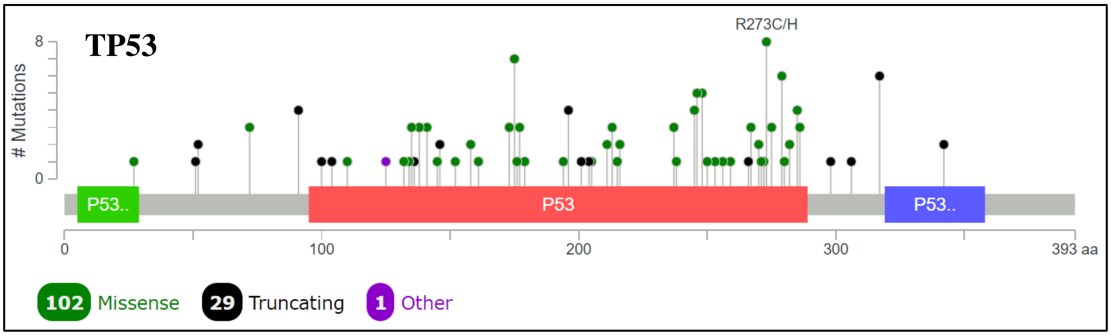
TP53
R273C/H
Mutations
8
0
P53..
P53
P53..
0
100
200
300
393 aa
102 Missense
29 Truncating
1 Other

Supplementary Figure 2: Kaplan-Meier survival curves of overall survival (ODS1 panel A and B; ODS2 panel C and D).

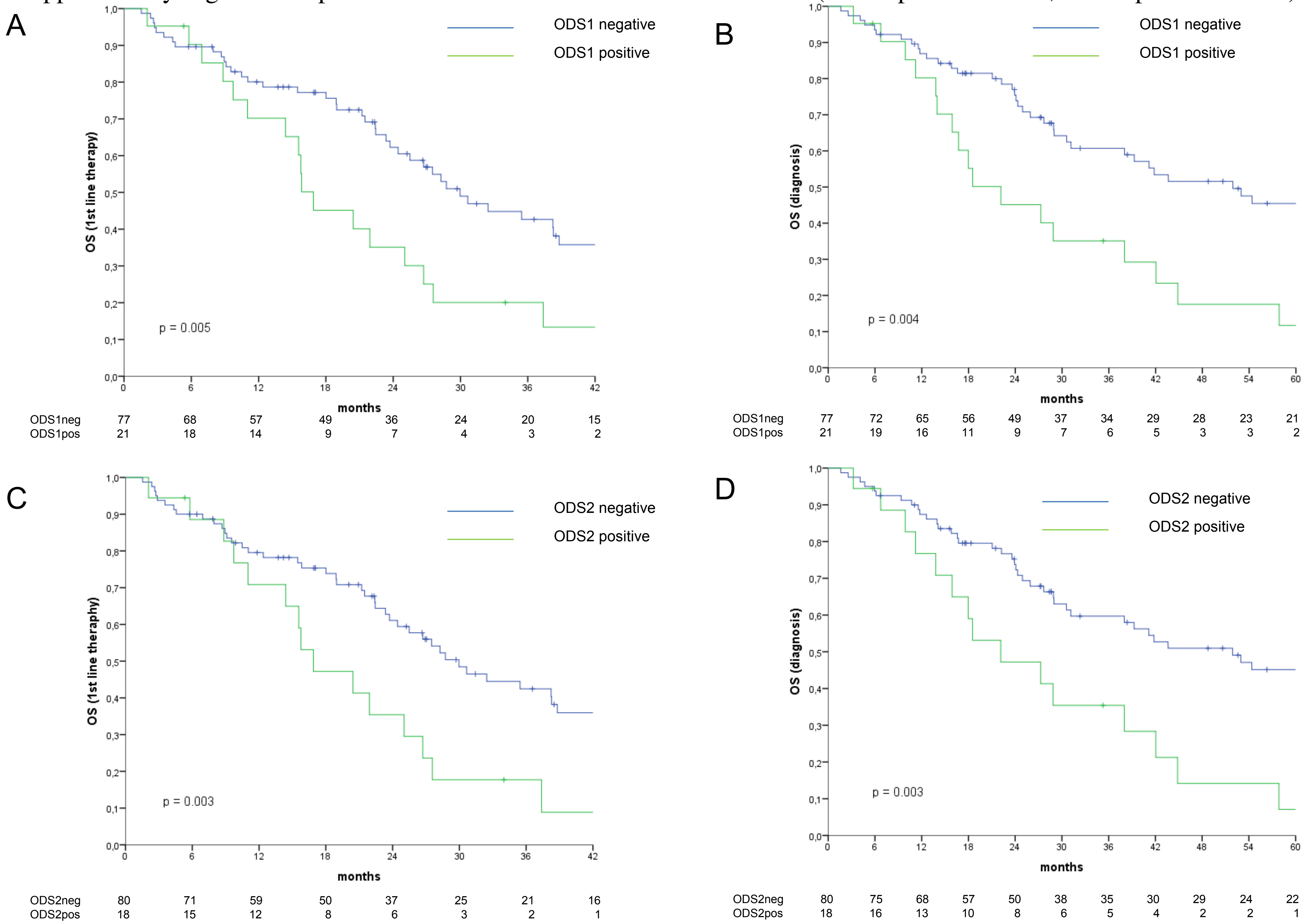

Supplementary Table 1: Mutation frequencies related to 16 CRC-associated genes observed in the present study (IRE-IFO) and in four publically available datasets (TCGA, DFCI, MSKCC, Genentech).

**GENE (PATHWAY)**	**IRE-IFO (N=98)**	TCGA (N=224)	DFCI (N=619)	MSKCC (N=69)	GENENTECH (N=72)
ACVR1B (TGF-β)	**1%**	6%	5%	0%	1.5%
AMER1 (WNT)	**7%**	11%	8%	9%	6%
APC (WNT)	**53%**	75%	57%	91%	40%
BRAF (RAS-MAPK)	**6%**	9%	20%	4%	8%
CTNNB1 (WNT)	**5%**	5%	6%	7%	7%
FBXW7 (WNT)	**21%**	17%	13%	9%	21%
KRAS (RAS-MAPK)	**58%**	42%	28%	55%	51%
MAP3K21 (RAS-MAPK)	**17%**	7%	4%	0%	0%
NRAS (RAS-MAPK)	**8%**	9%	4%	3%	3%
PIK3CA (PI3K-PTEN)	**33%**	20%	21%	22%	31%
PTEN (PI3K-PTEN)	**20%**	4%	8%	3%	6%
SMAD2 (TGF-β)	**8%**	7%	5%	0%	8%
SMAD4 (TGF-β)	**12%**	12%	11%	14%	24%
SOX9 (WNT)	**11%**	4%	10%	0%	3%
TCF7L2 (WNT)	**8%**	12%	7%	0%	8%
TP53 (p53 network)	**65%**	54%	50%	83%	56%

Supplementary Table 2: Statistically significant associations between the mutational status of 16 CRC-related genes and basal clinical features (N=98).

	**Stage at diagnosis**		**Chi2 Test p-value**
	**II-III N(%)**	**IV N(%)**	
**AMERwt**	29 (31.9)	62 (68.1)	0.047*
**AMERmut**	5 (71.4)	2 (28.6)	
**PTENwt**	23 (29.5)	55 (70.5)	0.032
**PTENmut**	11 (55.0)	9 (45.0)	
	**ECOG PS**		
	**0 N(%)**	**1-2 N(%)**	
**CTNNB1wt**	42 (45.2)	51 (54.8)	0.023*
**CTNNB1mut**	5 (100.0)	0 (0.0)	
**TP53wt**	11 (32.4)	23 (67.6)	0.024
**TP53mut**	36 (56.3)	28 (43.8)	
	**Number of Metastatic Sites**		
	**1 N(%)**	**≥2 N(%)**	
**FBXW7wt**	36 (46.8)	41 (53.2)	0.017
**FBXW7mut**	16 (76.2)	5 (23.8)	
	**Side**		
	**Left N(%)**	**Right + Transverse N(%)**	
**KRASwt**	28 (68.3)	13 (31.7)	0.039
**KRASmut**	27 (47.4)	30 (52.6)	
**TP53wt**	14 (41.2)	20 (58.8)	0.030
**TP53mut**	41 (64.1)	23 (35.9)	

* Fisher's Exact Test

Supplementary Table 3: Mutational status of the genes included in the OncoDriver signature 1 and 2 (ODS1 and ODS2).

GENE<br><br>SIGNATURE	TP53	APC	AMER1	TCF7L2	FBXW7	SOX9	CTNNB1
ODS1	MUT	MUT	WT	WT	WT	Not Included	Not Included
ODS2	MUT	MUT	WT	WT	WT	WT	WT

Supplementary Table 4: Univariate and multivariate Cox regression models for progression-free survival (N=98). The multivariate Cox models were obtained by adjusting for all the variables tested in univariate analysis.

		**Univariate Cox regression model**		**Multivariate Cox regression model (ODS1 pos vs neg)**		**Multivariate Cox regression model (ODS2 pos vs neg)**	
		**HR (95%CI)**	**p-value**	**HR (95%CI)**	**p-value**	**HR (95%CI)**	**p-value**
**ODS1**	**Pos vs Neg**	2.42 (1.44-4.06)	0.001	2.16 (1.26-3.70)	0.005		
**ODS2**	**Pos vs Neg**	2.76 (1.59-4.80)	<0.001			2.56 (1.45-4.51)	0.001
**Gender**	**Female vs Male**	0.79 (0.51-1.24)	0.315	0.84 (0.53-1.35)	0.478	0.82 (0.51-1.30)	0.399
**ECOG PS**	**1-2 vs 0**	1.25 (0.82-1.89)	0.293	1.24 (0.81-1.90)	0.329	1.19 (0.78-1.83)	0.413
**Side**	**DX+TV vs SX**	0.93 (0.61-1.41)	0.730	0.90 (0.57-1.44)	0.675	0.87 (0.55-1.39)	0.559
**Targeted Agent in first line therapy**	**Yes vs No**	0.86 (0.57-1.31)	0.498	0.80 (0.51-1.27)	0.354	0.83 (0.53-1.31)	0.434
**Surgery for metastatic disease**	**Yes vs No**	0.63 (0.40-1.01)	0.054	0.60 (0.37-0.97)	0.039	0.59 (0.36-0.95)	0.032
**Number of metastatic sites**	**2-3 vs 1**	1.75 (1.14-2.68)	0.010	1.84 (1.17-2.89)	0.009	1.91 (1.21-3.00)	0.005

Supplementary Table 5: Uni- and multivariate Cox regression models for overall survival (N=98). The multivariate Cox models were obtained by adjusting for all the variables tested in univariate analysis.

		**Univariate Cox regression model**		**Multivariate Cox regression model (ODS1 pos vs neg)**		**Multivariate Cox regression model (ODS2 pos vs neg)**	
		**HR (95%CI)**	**p-value**	**HR (95%CI)**	**p-value**	**HR (95%CI)**	**p-value**
**ODS1**	**Pos vs Neg**	2.21 (1.26-3.88)	0.006	1.96 (1.07-3.59)	0.030		
**ODS2**	**Pos vs Neg**	2.34 (1.31-4.20)	0.004			2.22 (1.18-4.16)	0.013
**Gender**	**Female vs Male**	0.79 (0.47-1.33)	0.369	0.63 (0.36-1.11)	0.109	0.61 (0.35-1.08)	0.091
**Stage at diagnosis**	**IV vs II-III**	1.24 (0.74-2.08)	0.412	1.23 (0.72-2.12)	0.445	1.20 (0.70-2.05)	0.515
**ECOG PS**	**1-2 vs 0**	2.12 (1.28-3.52)	0.003	1.89 (1.10-3.25)	0.021	1.83 (1.06-3.14)	0.030
**Side**	**DX+TV vs SX**	1.07 (0.66-1.76)	0.777	0.86 (0.50-1.49)	0.588	0.83 (0.48-1.42)	0.491
**Second line therapy**	**Yes vs No**	0.29 (0.17-0.48)	<0.001	0.17 (0.09-0.32)	<0.001	0.16 (0.09-0.31)	<0.001
**Surgery for metastatic disease**	**Yes vs No**	0.58 (0.34-0.99)	0.046	0.49 (0.28-0.88)	0.016	0.50 (0.28-0.90)	0.020
**Number of metastatic sites**	**2-3 vs 1**	1.40 (0.85-2.33)	0.188	1.83 (1.05-3.17)	0.031	1.85 (1.07-3.20)	0.028
**Targeted Agents**	**Yes vs No**	0.66 (0.40-1.07)	0.094	0.83 (0.48-1.43)	0.503	0.81 (0.47-1.40)	0.449
